# Stunting among children aged 24–59 months and associations with sanitation, enteric infections, and environmental enteric dysfunction in rural northwest Ethiopia

**DOI:** 10.1038/s41598-022-23981-5

**Published:** 2022-11-11

**Authors:** Zemichael Gizaw, Alemayehu Worku Yalew, Bikes Destaw Bitew, Jiyoung Lee, Michael Bisesi

**Affiliations:** 1 Department of Environmental and Occupational Health and Safety, Institute of Public Health, College of Medicine and Health Sciences, University of Gondar, Gondar, Ethiopia; 2 Addis Continental Institute of Public Health, Addis Ababa, Ethiopia; 3 Global One Health Initiative (GOHi), The Ohio State University, Columbus, OH USA; 4 School of Public Health, Addis Ababa University, Addis Ababa, Ethiopia; 5 Division of Environmental Health Sciences, College of Public Health, The Ohio State University, 1841 Neil Avenue, Columbus, OH 43210 USA; 6 Department of Food Science and Technology, The Ohio State University, Columbus, OH USA

**Keywords:** Diseases, Gastrointestinal diseases, Infectious diseases, Nutrition disorders

## Abstract

Stunting is a public health issue of global concern. Despite, poor sanitation, diarrhea, parasitic infections, and environmental enteric dysfunction (EED) are associated with stunting, their link is poorly understood and has not been investigated in Ethiopia. This study was conducted to assess the associations of stunting with sanitation, enteric infections, and EED among children aged 24–59 months in rural northwest Ethiopia. A community-based cross-sectional study was conducted among 224 randomly selected children aged 24–59 months in rural areas of the east Dembiya district. We collected information on household food insecurity and dietary diversity using pre-tested questionnaires adopted from the food and nutrition technical assistance (FANTA) project. We used height-for-age-z score (HAZ) to define stunting. We also used the data collected to measure the environmental exposures of children to intestinal parasitic infections and fecal biomarkers of EED. A multivariable binary logistic regression model was used to assess the association of stunting with sanitation, enteric infections, and EED. Of the 224 children, 33% (95% CI 27, 39%) were stunted. Stunting in children was significantly associated with poor dietary intake (AOR 3.0, 95% CI 1.2, 7.3), open defecation practice (AOR 3.0, 95% CI 1.2, 7.9), presence of animal excreta in the living environment (AOR 3.4, 95% CI 1.2, 9.9), *E. coli* contamination of drinking water (AOR 4.2, 95% CI 1.1, 15.3), diarrheal disease incidence (AOR 3.4, 95% CI 1.5, 7.7), intestinal parasites in children (AOR 3.3, 95% CI 1.3, 8.8), and higher EED disease activity scores (AOR 2.9, 95% CI 1.2, 6.7). One-third of the children in the study area were stunted and this high prevalence of stunting was associated with poor dietary intake, poor hygiene and sanitation conditions, enteric infections, and EED. Thus, stunting can be prevented by improving sanitation and hygienic conditions to prevent repeated enteric infections in children and by promoting dietary diversity of children.

## Introduction

Poor sanitation, enteric infections, environmental enteric dysfunction, and stunting are public health issues of global concern and failure to provide sanitation and nutrition services in the twenty-first century is the greatest development failure^1–3^ that leads to diseases^4–6^ and stifles social and economic development by negatively impacting health, education, and livelihoods and results stunted growth^7–9^. Stunting is defined as a deficit in height relative to a child’s age^10^. Children in the developing world are the most impacted group by stunting^5^. According to the 2018 United Nations Children’s Fund (UNICEF) report, 149 million (21.9%) children under 5 years of age across the globe and 58.8 million (30%) in Africa are stunted^11^ and in Ethiopia 38% children under 5 years of age were stunted in 2019^12^. Stunting in children could lead to impaired physical development and have a long-term effect on cognitive development, educational performance, and economic productivity in adulthood and on maternal reproductive outcomes^13,14^. Maternal stunting could result high risks to the survival, health and development of her offspring. Decreased maternal stature is associated with an increased risk of underweight and stunting among offspring. Maternal stunting can restrict uterine blood flow and growth of the uterus, placenta, and fetus. Intrauterine growth restriction (IUGR) is associated with many adverse fetal and neonatal outcomes^15^.

Traditionally, stunting was believed to be caused by lack of food^16^. Adequate and nutritious foods are necessary to nourish children but not enough. Only a small portion of stunting can be solved by nutrition interventions^17^. Research findings indicated that stunting occurs even among well-fed children^18^, which showed that there are other factors, such as poor WASH conditions which are linked to stunting^19,20^. Children who live without access to basic WASH services do not grow as children with access to adequate sanitation^21,22^. Unhygienic disposal of human and animal excrement causes fecal contamination of the living environment^23–26^. This results a continuous exposure of children to enteropathogens through the ingestion of contaminated food, water, and even soil^27–29^. Microorganisms like rotavirus, adenovirus, and astrovirus cause limited mucosal disturbances. Enterotoxigenic *E. coli* (ETEC) causes secretory diarrhea with only limited mucosal changes. Others, such as Campylobacter, Shigella, Salmonella, enteroaggregative *E. coli* (EAEC), enteropathogenic *E. coli* (EPEC), and enteroinvasive *E. coli* (EIEC) are enteroinvasive or cause extensive mucosal disruption^30^. Generally, enteropathogens can cause chronic gut inflammation and morphological abnormalities in the small intestine such as blunted villi and crypt hyperplasia^27,31^, which can cause bacterial translocation, systemic inflammation, metabolic changes, increased permeability, and nutrient malabsorption, growth faltering. This process is known as EED, is a poorly understood condition that has the potential to affect child growth, health, and development in low and middle-income countries (LMICs)^32^. The pathogenic pathways in EED is characterized by (i) gut inflammation, (ii) gut leakiness/permeability, (iii) microbial translocation, (iv) dysbiosis, (v) systemic inflammation, and (vi) nutrient malabsorption^33,34^.

Enteric infections and EED tie poor WASH to stunting^35,36^. However, the link between poor WASH, enteric infections, EED, and stunting is poorly understood^37^ and it has not been investigated in Ethiopia. Accordingly, this study was conducted to assess the associations of stunting with sanitation, enteric infections, and EED among children aged 24–59 months in rural northwest Ethiopia.

## Methods

This community-based cross-sectional study is part of a large-scale study conducted in rural northwest Ethiopia during May–June 2021 to assess environmental exposures of children to enteric infections and fecal biomarkers of EED in children aged 24–59 months. The method has been described in more detail elsewhere^38,39^. The sample size was determined using double proportion population formula with the following assumptions: proportion of stunting with enteric infection (p_1_) = 38.5%, proportion of stunting with no enteric infection (p_2_) = 61.5%^40^, Z_α/2_ at type I error of 5% = 1.96, *Z*_*β*_ at 80% power = 0.842, and an allocation ratio of 1:1. Therefore, the sample size was determined to be 71 in each group. After considering a design effect of 1.5 and 5% non-response rate, the final sample size in each group was found to be 112, leading to a total of 224 study subjects. In the current study, we randomly selected 224 out of 235 children included in a study done to measure fecal biomarkers of EED^39^ using a simple random sampling technique, i.e., computer-based random number generator to further analyze the data for nutritional assessments.

### Food access and nutrition assessment

Household food insecurity was assessed using a pre-tested food insecurity assessment questionnaire adopted from the FANTA project, version 3, and households were classified as food secure, mildly food insecure, moderately food insecure, and severely food insecure^41^. The FANTA household food insecurity access scale generic questions that have been used in several countries and appear to distinguish the food secure from the insecure households across different cultural contexts^41^.

Dietary intake of the children over a period of 24 h was assessed using a pre-tested dietary diversity questionnaire adopted from the FANTA project^42^. The caregivers were asked whether the children had eaten foods from the seven main food groups in a 24-h period. The food groups assessed were: (a) grains, roots, or tubers; (b) vitamin A-rich plant foods; (c) other fruits and vegetables; (d) flesh foods (meat, poultry, fish, and seafood); (e) eggs; (f) pulses, legumes, or nuts; and (g) milk and milk products. Dietary diversity score (DDS) was used to qualitatively assess the dietary intake of children. Poor dietary diversity was defined as a child with a DDS of less than four^43^. Height was measured using the seca vertical height scale (German, serial No. 0123) standing upright in the middle of the board. The child’s head, shoulders, buttocks, knees, and heels touch the vertical board and the height-for-age-z score (HAZ) was calculated using WHO Anthro software, and the values of each child were compared with the WHO Multicenter Growth Reference Standard. Trained data collectors who have BSc degree in Public Health, Nursing, and Environmental Health performed the measurement and data collection.

### Measurement of study variables

Stunting among children is the primary outcome variable of this study and a child was categorized as stunted if his or her HAZ was less than − 2SD from the reference population. In addition, a child with HAZ less than − 3SD and between − 3 and − 2SD was considered severely and moderately stunted, respectively^44^.

Mouthing of soil-contaminated materials, fecal contamination of drinking water, fecal contamination of ready-to-eat foods, fecal contamination of courtyard soil, diarrheal disease, and intestinal parasites were the exposure variables for this study. Childhood diarrheal disease was defined as having three or more loose or watery stools within 24 h period^45^. A 2-week period of diarrheal disease in children was determined based on history from mothers or caregivers. Fecal contamination of the living environment (drinking water, ready-to-eat foods, and courtyard soil) was measured by detection of fecal indicator bacteria, i.e., *Escherichia coli* (*E. coli*) using membrane filter technique^46^. Sampling procedures of environmental samples and *E. coli* detection procedures are described elsewhere in more detail^38^. Intestinal parasites in children were measured by detecting ova of one or more intestinal parasites in stool samples of children using wet mount and Kato-Katz techniques^47^. The detailed procedures of stool sample collection and investigation of ova of parasites are described elsewhere in more detail^38^. Geophagy or mouthing of soil-contaminated materials is a repeated ingestion of nonfood substances (such as clays, yard soil), or large quantities of certain particular foods contaminated with soil^48^. Environmental enteric dysfunction was measured by three fecal biomarkers: Alpha-1-antitrypsin (AAT), myeloperoxidase (MPO), and neopterin (NEO). We used commercial ELISA kits to measure stool concentrations of MPO (Immundiagnostik AG, Germany), AAT (BioVendor, ImmuChorm, Germany), and NEO (GenWay Biotech Inc., USA). The use of a combination of biomarkers (AAT, MPO, and NEO) to measure EED is standardized in developing countries and the sensitivity and specificity of these biomarkers in predicting poor linear growth is well documented^49–51^. The measurement of fecal biomarkers of EED is described elsewhere in more detail^39^. Fecal concentrations of AAT, MPO, and NEO were categorized in to low (concentrations in first quartile), medium (concentrations in the interquartile range), or high (concentrations in fourth quartile). For each of the three biomarkers, 0 point was given for low concentrations, 1 point for medium concentrations, and 2 points for high concentrations and EED disease activity score was calculated as 2 × (AAT category) + 2 × (MPO category) + 1 × (NEO category)^49,52,53^.

### Statistical analysis

We used STATA version 14 (Stata Corp, College Station, TX, USA) to analyze the data. Multivariable binary logistic regression analysis was done to identify factors associated with stunting. Covariates for the adjusted model were selected using bivariate analysis on the basis of p-values < 0.2. In the adjusted model, statistically significant associations were declared based on adjusted odds ratio (AOR) with the corresponding 95% confidence interval (CI) and p-value < 0.05. Model fitness was checked using the Hosmer–Lemeshow model fitness test and we checked multicollinearity using variance inflation factors and we found no collinearity.

### Ethics approval and consent to participate

Ethical clearance was obtained from the Institutional Review Board of the University of Gondar (reference number: V/P/RCS/05/1933/2020). There were no risks due to participation and the collected data were used only for this research purpose with complete confidentiality. Written informed consent was obtained from mothers or caregivers. All the methods were carried out in accordance with relevant guidelines and regulations.

## Results

### Socio-demographic characteristics

Among the total 224 children included in this study, 50.9% of the participants in the study were females. The youngest and oldest children were 24 and 59 months old, respectively, with a mean age of 43 (± 13 SD) months. The highest proportion of children, 42%, were between the ages of 49 and 59 months. About one-tenth (11%) of mothers or caregivers attended post-secondary education. Moreover, 60% of the households had a family size of below five and 69% of the households owned livestock (Table 1).

**Table 1:** Sociodemographic characteristics of mothers and children (n = 224) dyad in rural areas of east Dembiya district, northwest Ethiopia, May–June 2021.

Socio-demographic characteristics	Frequency (n)	Percent (%)
**Sex**		
Male	110	49
Female	114	51
**Age in month**		
24–36	52	23
37–48	77	34
49–59	95	42
**Education status of mothers**		
Cannot read and write	85	38
Can read and write	17	8
Primary education	46	21
Secondary education	51	23
Post-secondary education	25	11
**Family size**		
≤ 5	135	60
> 5	89	40
**The household has livestock**		
Yes	155	69
No	69	31

### Hygiene and sanitation conditions

The results showed that 69% of the households defecated in the open field. We found animal excreta in the courtyards in 76% of the households during the spot-check observation. Furthermore, *E. coli* was detected in 81%, 70%, and 67% of courtyard soil, drinking water, and ready-to-eat food samples, respectively. We also observed that 71% of the children mouthed soil-contaminated materials (Table 2).

**Table 2:** Hygiene and sanitation conditions of children (n = 224) in rural areas of east Dembiya district, northwest Ethiopia, May – June 2021.

Hygiene and sanitation conditions	Frequency (n)	Percent (%)
**Defecation practice of the household**		
Open field	154	69
Sanitary latrine	70	31
**Animal excreta in the living environment**		
Yes	170	76
No	54	24
***E. coli* detected in courtyard soil samples**		
Yes	182	81
No	42	19
***E. coli* detected in drinking water samples**		
Yes	157	70
No	67	30
***E. coli* detected in ready-to-eat food samples**		
Yes	149	66
No	75	34
**Children mouthed soil-contaminated materials**		
Yes	159	71
No	65	29

*E. coli*: *Escherichia coli.*

### Enteric infections and environmental enteric dysfunction in children

In the current study, 61% of the children had one or more intestinal parasitic infections and 31% of the mothers or caregivers reported that their children had diarrhea in a 2 week period prior to the survey and 9% of the children had both diarrheal disease and intestinal parasitic infections. Moreover, the concentration of Alpha-1-antitrypsin (AAT) was elevated among 67% of the children compared with the normal concentration, i.e., < 270 μg/ml. Similarly, the concentrations of Myeloperoxidase (MPO) and Neopterin (NEO), respectively were elevated in 72% and 79% of the children compared with concentrations considered normal, i.e., < 2000 ng/ml for MPO and < 70 nmol/l for NEO. Moreover, 42% of the children had high EED disease activity scores (above the median score of 5), indicating that the concentrations of fecal biomarkers in these children are elevated (Table 3).

**Table 3:** Distribution of fecal biomarkers of EED among children (n = 224) in rural areas of northwest Ethiopia, May to June 2021.

Fecal biomarkers of EED	Frequency (n)	Percent (%)
**AAT (μg/ml)**		
Normal	74	33
Elevated	150	67
**MPO (ng/ml)**		
Normal	62	28
Elevated	162	72
**NEO (nmol/l)**		
Normal	47	21
Elevated	177	79
**EED disease activity scores**		
Low	131	59
High	93	41

*μg/ml* microgram per milliliter, *AAT* alpha-1-antitrypsin, *EED* environmental enteric dysfunction, *MPO* myeloperoxidase, *NEO* neopterin, *ng/ml* nanogram per milliliter, *nmol/l* nanomoles per milliliter.

### Food access and nutrition status of children

In the study area, 47% of the households were food insecure, out of which 19% were severely insecure. The minimum dietary score (children who consumed foods from four or more food groups) was 69%. Grains, roots, or tubers (97%); pulses, legumes, or nuts (90%); and milk and milk products (74%) were the most commonly consumed food groups. Of the 224 children, 73 had HAZ less than − 2SD. The prevalence of stunting was, therefore, found to be 33% (95% CI 27, 39%), out of which 5% and 27% were severely and moderately stunted (Table 4).

**Table 4:** Nutritional status of children (n = 224) in rural areas of northwest Ethiopia, May to June 2021.

Food access and nutrition status	Frequency (n)	Percent (%)
**Household food security**		
Food secure	118	53
Mildly food insecure	11	5
Moderately food insecure	53	24
Severely food insecure	42	19
**Minimum dietary intake**		
< 4 food groups	70	31
≥ 4 food groups	154	69
**Food groups**		
Grains, roots, or tubers	217	97
Pulses, legumes, or nuts	202	90
Milk and milk products	165	74
Flesh foods (meat, poultry, and fish)	125	56
Vitamin A-rich plant foods	73	33
Other fruits and vegetables	80	36
Eggs	45	20
**Stunting**		
Not stunted	151	67
Moderately stunted	61	27
Severely stunted	12	5

**Figure 1:**
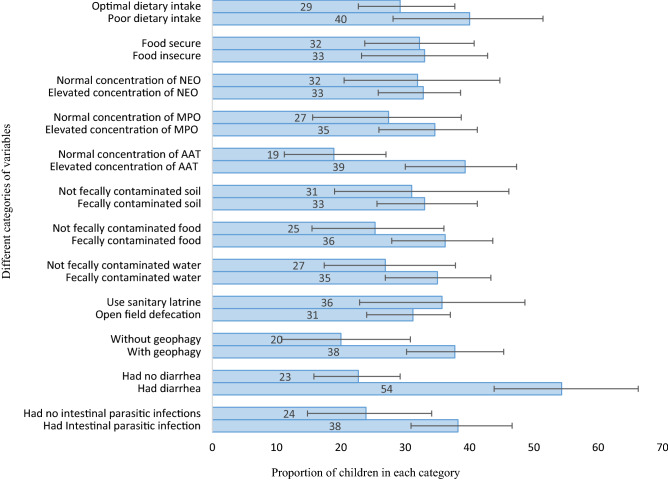
Proportion of stunted children (n = 224) grouped in to different variables in rural areas of northwest Ethiopia, May to June 2021. Error bars show the 95% CI of prevalence of stunting. *AAT* alpha-1-antitrypsin, *MPO* myeloperoxidase, *NEO* neopterin.

The prevalence of stunting in various groups of children is illustrated in Fig. 1. Children with intestinal parasitic infections, for example, had a higher prevalence of stunting than children without such infections. Similarly, when comparing children with elevated fecal AAT, MPO, and NEO concentrations to children with normal concentrations, the prevalence of stunting was very high.

### Factors associated with stunting

Child age, sex of a child, education status of mothers, household food security, dietary diversity, defecation practice of the household, animal excreta in the living environment, mouthing of soil-contaminated materials, *E. coli* contamination of drinking water, *E. coli* contamination of courtyard soil, *E. coli* contamination of ready-to-eat foods, childhood diarrhea, intestinal parasites in children, and EED disease activity scores were entered in to the bivariate analysis. In the final model, only dietary diversity, defecation practice of the household, animal excreta in the living environment, *E. coli* contamination of drinking water, childhood diarrhea, intestinal parasites in children, and EED disease activity scores were significantly associated with stunting among children.

This study depicted that children who had poor dietary intake had higher odds of stunting compared with children who had good dietary intake (AOR 3.0, 95% CI 1.2, 7.3). Stunting among children in the study area was also significantly associated with WASH conditions. For instance, open defecation practice (AOR 3.0, 95% CI 1.2, 7.9) and presence of animal excreta in the living environment (AOR 3.4, 95% CI 1.2, 9.9) was associated with higher odds of stunting among children. Similarly, *E. coli* contamination of drinking water was significantly associated with 4.2 times higher odds of stunting among children (AOR 4.2, 95% CI 1.1, 15.3). Moreover, enteric infections and EED were statistically associated with stunting among children in the study area. The odds of stunting was 3.4 times higher among children who had diarrhea compared with children who had no diarrhea (AOR 3.4, 95% CI 1.5, 7.7). Similarly, the odds of stunting was 3.3 times higher among children who had one or more intestinal parasites compared with children who had no parasites (AOR 3.3, 95% CI 1.3, 8.8). Higher EED disease activity scores was also significantly associated with higher odds of stunting among children compared with low EED disease activity scores (AOR 2.9, 95% CI 1.2, 6.7) (Table 5).

**Table 5:** Factors associated with stunting among children (n = 224) in rural areas of northwest Ethiopia, May to June 2021.

Covariates	Stunting in children		COR with 95% CI	AOR with 95% CI
	Yes	No		
**Household food security**				
Food secured	29	89	1.0	1.0
Food insecured	44	62	2.2 (1.2, 3.9)	0.9 (0.4, 2.1)
**Minimum dietary intake**				
< 4 food groups	27	43	1.5 (0.8, 2.7)	3.0 (1.2, 7.3)*
≥ 4 food groups	46	108	1.0	1.0
**Mouthing of soil-contaminated materials**				
Yes	60	99	2.4 (1.2, 4.8)	1.1 (0.4, 2.7)
No	13	52	1.0	1.0
**Defecation practice of the household**				
Open field defecation	61	93	3.2 (1.6, 6.4)	3.0 (1.2, 7.9)*
Use latrine	12	58	1.0	1.0
**Animal excreta in the living environment**				
Yes	67	103	5.2 (2.1, 12.8)	3.4 (1.2, 9.9)*
No	6	48	1.0	1.0
***E. coli* detected in courtyard soil**				
Yes	66	116	2.9 (1.2, 6.8)	0.9 (0.2, 3.0)
No	7	35	1.0	1.0
***E. coli* detected in drinking water**				
Yes	69	88	12.4 (4.3, 35.6)	4.2 (1.1, 15.3)*
No	4	63	1.0	1.0
***E. coli* detected in ready-to-eat foods**				
Yes	67	82	9.4 (3.8, 23.0)	1.4 (0.4, 4.5)
No	6	69	1.0	1.0
**Childhood diarrhea**				
Yes	34	36	2.8 (1.5, 5.0)	3.4 (1.5, 7.7)**
No	39	115	1.0	1.0
**Intestinal parasites**				
Yes	64	72	7.8 (3.6, 16.8)	3.3 (1.3, 8.8)*
No	9	79	1.0	1.0
**EED disease activity scores**				
Low	22	109	1.0	1.0
High	51	42	6.0 (3.3, 11.1)	2.9 (1.2, 6.7)**

*Statistically significant at p < 0.05, **statistically significant at p < 0.01, Hosmer–Lemeshow test = 0.187.

*E. coli*: *Escherichia coli*, EED: Environmental enteric dysfunction; COR: Crude odds ratio; AOR: Adjusted odds ratio.

High EED disease activity score: EED scores above the median score of 5. Low EED disease activity score: EED scores below the median score of 5.

## Discussion

This community-based cross-sectional study was conducted to assess stunting and its associations with environmental factors (hygiene and sanitation), and its related host factors (enteric infections and EED) among children aged 24–59 months in rural northwest Ethiopia. We found that *E. coli* was detected in 81%, 70%, and 67% of courtyard soil, drinking water, and ready-to-eat food samples, respectively. Ova of one or more of intestinal parasites were detected in 61% of the children and 31% of the children had diarrhea in a 2 week period prior to the survey. The concentration of AAT among 67%, myeloperoxidase among 72%, and neopterin among 79% of the stool samples were above the normal values and 42% of the children had high EED disease activity scores. Moreover, 33% (95% CI 27, 39%) of the children were stunted.

The prevalence of stunting reported in this study is comparable with findings of similar studies conducted in different parts of Ethiopia, such as in west Guji zone (32%)^54^, southwest Ethiopia (33%)^55^, Mizan Aman town (35%)^56^, and Hosanna (35%)^57^. Similarly, this finding is comparable with findings of studies in rural Sierra Leone (32%)^58^, rural Bangladesh (27%)^59^, and rural Zambia (35%)^60^. Prevalence of stunting in the current study is also lower than findings of other studies in different parts of Ethiopia, such as in Albuko district (39%)^61^, Dembia district (46%)^62^, Angolela Tera district (39%)^63^, Boricha district (49%)^64^, Arba Minch (48%)^65^, Bule Hora district (48%)^66^, Lalibela town (47%)^67^, and Enticho town (47%)^68^. Compared with other developing countries, the prevalence reported in this study was lower studies conducted among children under 5 years of age in a rural area of Maharashtra, India (42%)^69^, rural Rwenzori Sub-Region of Western Uganda (45%)^70^, Manyovu, Buhigwe District of Tanzania (43%)^71^, and rural Community in Kaduna State of North Western Nigeria (60%)^72^. Moreover, the current study reported higher prevalence of stunting compared with findings of studies in Hawassa Zuria district (27%)^73^, Sodo Zuria district (25%)^74^, Debretabor town (23%)^75^, and Wolayta Sodo (22%)^76^. The prevalence of stunting reported in the current study is also higher than reports of a study in Bandja village of Cameroon (16%)^77^.

The high prevalence of stunting among children in the studied region may be explained by poor hygiene and sanitation conditions, a high burden of enteric infections, and gut inflammation of children in the area. The association between poor WASH and stunting is due to the microbial contamination of soil, water, and food and subsequent acquisition of enteric infections such as helminthiases and diarrheal disease by children^78–80^, which have been reported to be associated with stunting^81,82^. Intestinal worms and other enteropathogens can cause chronic gut inflammation and morphological abnormalities in intestine lead to increased intestinal permeability with subsequent bacterial translocation and immune stimulation^27,31^, increased energy expenditure due to systemic inflammation^27,31^, metabolic alterations^27,31^, reduced absorption capacity of the intestine and altered nutrient absorption^27,31^, and nutrient malabsorption or decreased nutrient intake^27,31^.

Moreover, the high prevalence of stunting among children in the study area may be associated with poor dietary intake, which is in line with findings of other studies^83–85^. Insufficient dietary intake of nutrients can irreversibly harm children’s rapidly growing bodies and brains, limiting their potential to grow^86^. Receiving an inadequately diversified diet may predispose children to opportunistic infections and severe illnesses^87,88^. As a result, the world health organization has recommended that an infant should receive at least four food groups out of seven in order to maintain proper growth and development during this critical period^89^.

As a strength, we assessed EED using fecal AAT, MPO, and NEO since the use of these three fecal biomarkers in combination can better explain EED than any single biomarker^49,50^. Moreover, to effectively answer the research question, we used a combination of methods, including observation of environmental sanitation and child behaviors that result in a high risk of infection; household survey; laboratory investigation of water, food, soil, and stool samples; food security and dietary assessment; and anthropometric assessment. However, although the use of fecal biomarkers permits assessment of intestinal/systemic inflammation and/or intestinal epithelial barrier dysfunction, the main limitation to their use is that they are not specific for EED because they correlate with prevalence, activity, and/or severity of various other gastrointestinal diseases and the fecal biomarkers may not be reflective for small bowel activity^90^. Furthermore, HAZ is inappropriate to measure changes in linear growth over time because they are constructed using standard deviations from cross-sectional data. The self-reported nature of food insecurity and dietary diversity data, as well as a 4-week and a 24-h recall period, make the data prone to social desirability and recall biases. In addition to social desirability and recall biases, there was another significant limitation to this study. A 24-h food consumption assessment is likely to be subjected to normal day-to-day variability, and it also ignores the amount of food consumed. Despite this, the methods used in this study have been widely implemented and validated. This study's limitations are not unique. Others have noted the limitations of self-reported food insecurity and dietary diversity data collection methods^91,92^. We attempted to reduce bias by assuring respondents of confidentiality and privacy, as well as informing them that their responses to the questions would have no bearing on their eligibility for aid. Moreover, the use of bivariate p-values to select candidate variables for the adjusted model could lead to the incorrect exclusion of a potential confounder and hence led to an inadequate adjustment for confounding.

## Conclusion

One-third of the children in the study area had stunted growth and stunting in the study area was associated with poor dietary intake, poor hygiene and sanitation conditions, enteric infections, and EED. This suggests that lack of access to adequate and nutritious foods is not the only cause of stunting among children in the studied region. Thus, stunting can be prevented by improving sanitation and hygienic conditions to prevent repeated enteric infections in children and by promoting dietary diversity of children.

## Data Availability

Data will be made available upon requesting the primary author, i.e., ZG.

## Abbreviations

μg/ml: Microgram per milliliter
AAT: Alpha-1-antitrypsin
CI: Confidence interval
DDS: Dietary diversity score
EED: Environmental enteric dysfunction
*E. coli*: *Escherichia coli*
FANTA: Food and nutrition technical assistance
HAZ: Height-for-age-z score
MPO: Myeloperoxidase
NEO: Neopterin
ng/ml: Nanogram per milliliter
nmol/l: Nanomoles per milliliter
SD: Standard deviation
UNICEF: United Nations Children’s Fund
WASH: Water, sanitation and hygiene
WHO: World Health Organization
